# Immunotherapy may protect cancer patients from SARS-CoV-2 infection: a single-center retrospective analysis

**DOI:** 10.1186/s12967-021-02798-2

**Published:** 2021-03-31

**Authors:** Maria Antonietta Isgrò, Maria Grazia Vitale, Egidio Celentano, Flavia Nocerino, Giuseppe Porciello, Marcello Curvietto, Domenico Mallardo, Concetta Montagnese, Luigi Russo, Nicoletta Zanaletti, Antonio Avallone, Matilde Pensabene, Michelino De Laurentiis, Sara Centonze, Sandro Pignata, Lucia Cannella, Alessandro Morabito, Francesco Caponigro, Gerardo Botti, Giuseppe Valentino Masucci, Diana Giannarelli, Ernesta Cavalcanti, Paolo Antonio Ascierto

**Affiliations:** 1 Division of Laboratory Medicine, Istituto Nazionale Tumori – IRCCS – Fondazione G. Pascale, Naples, Italy; 2 Unit of Melanoma, Cancer Immunotherapy and Innovative Therapy, Istituto Nazionale Tumori – IRCCS – Fondazione G. Pascale, Naples, Italy; 3 Epidemiology and Biostatistics Unit, Istituto Nazionale Tumori – IRCCS – Fondazione G. Pascale, Naples, Italy; 4 Abdominal Oncology Division, Istituto Nazionale Tumori – IRCCS – Fondazione G. Pascale, Naples, Italy; 5 Department of Breast and Thoracic Oncology, Istituto Nazionale Tumori – IRCCS – Fondazione G. Pascale, Naples, Italy; 6 Department of Urology and Gynecology, Istituto Nazionale Tumori – IRCCS – Fondazione G. Pascale, Naples, Italy; 7 Department of Muscle-Skeletal Oncology, Istituto Nazionale Tumori – IRCCS – Fondazione G. Pascale, Naples, Italy; 8 Department of Thoracic Medical Oncology, Istituto Nazionale Tumori – IRCCS – Fondazione G. Pascale, Naples, Italy; 9 Head and Neck Medical Oncology Unit, Istituto Nazionale Tumori – IRCCS – Fondazione G. Pascale, Naples, Italy; 10 Scientific Directorate, Istituto Nazionale Tumori – IRCCS – Fondazione G. Pascale, Naples, Italy; 11 Department of Oncology-Pathology, Karolinska Institutet, Cancer Centrum Karolinska, 171 76 Stockholm, Sweden; 12 Biostatistic Unit, Istituto Nazionale Tumori Regina Elena, IRCCS, Rome, Italy

**Keywords:** Immunotherapy, Chemotherapy, SARS-CoV-2 infections, ICIs, SARS-CoV-2 seropositivity

## Abstract

Coronavirus disease 2019 (COVID-19) global pandemic has created unique challenges to healthcare systems throughout the world. Ensuring subjects’ safety is mandatory especially in oncology, in consideration of cancer patients’ particular frailty. We examined the proportion of severe acute respiratory syndrome coronavirus 2 (SARS-CoV-2) IgM and/or IgG positive subjects in three different groups from Istituto Nazionale Tumori – IRCCS “Fondazione G. Pascale” in Naples (Campania region, Italy): cancer patients treated with Innovative Immunotherapy (Immune Checkpoint Inhibitors, ICIs), cancer patients undergoing standard Chemotherapies (CHTs) and healthcare providers. 9 out of 287 (3.1%) ICIs patients resulted positive, with a significant lower percentage in respect to CHTs patients (39 positive subjects out of 598, 6.5%) (p = 0.04). There was no statistically significant difference between ICIs cohort and healthcare providers, 48 out of 1050 resulting positive (4.6%). Performing a Propensity Score Matching based on gender and tumor stage, the effect of treatment on seropositivity was analyzed through a regression logistic model and the ICIs treatment resulted to be the only protective factor significantly (p = 0.03) associated with positivity (odds ratio—OR: 0.41; 95% confidence interval—CI 0.18–0.91). According to these preliminary data, ICIs would appear to be a protective factor against the onset of COVID-19 infection.

## Introduction

During coronavirus disease 2019 (COVID-19) global pandemic, the oncologist community is debating about two main issues: the first is to guarantee continuity of care for cancer patients, the second is about patients’ safety. A recent paper published in Nature Medicine has stated that determining the incidence of COVID-19 through the use of large-scale serological testing is a priority, caring for patients with cancer [1].

A pooled meta-analysis of 11 retrospective studies reported that nearly 2.0% of patients with COVID-19 were affected by cancer, showing an increased susceptibility to COVID-19 infection [2]. Nevertheless, data from a French trial carried out by Barlesi et al. from Gustave Roussy Cancer Center involving 137 cancer patients diagnosed with severe acute respiratory syndrome coronavirus 2 (SARS-CoV-2) showed a rate of infection similar to that of global population [3].

Data regarding COVID-19 infection severity in cancer patients are discordant and limited to date. First reports recorded more severe symptoms and worse outcomes in these patients [4, 5]. Zhang et al. reported that cancer seems to be associated most frequently with severe SARS-CoV-2 infection in a subset of lung cancer patients [6]. On the contrary, Barlesi et al. didn’t find any evidence that COVID-19 is more lethal or aggressive in cancer patients [3].

Another concern is about the relationship between type of oncological therapy and the development of COVID-19 infection. While standard Chemotherapies (CHTs) are generally associated with immunosuppression and increased risk of infection, the role of Innovative Immunotherapy (Immune Checkpoint Inhibitors, ICIs) in promoting severe SARS-CoV-2 infection is in fact source of debate. As we know, Innovative Immunotherapy, based on ICIs, activates the immune system against cancer, but the inflammatory storm of the activated immune system could be directed against other organs (interstitial pneumonia occurs in 2.5–5% of ICIs in monotherapy and in 7–10% of combination therapy). The risk of overlapping syndromes due to SARS-CoV-2 infection with similar pathogenesis is theoretic, but it cannot be excluded. Few data support this idea. A retrospective study of Memorial Sloan Kettering Cancer Center enrolled 423 patients with COVID-19 infection and concomitant cancer. Among these patients 31 (7%) were treated with ICIs. The study demonstrated that ICIs treatment within 90 days is a predictor for hospitalization and severe disease: OR (odds ratio) of 2.53 (95% confidence interval—CI 1.18–5.67), p = 0.017 in univariate analysis; OR of 2.84 (95% CI 1.24–6.72), p = 0.013 in multivariate analysis [7]. Otherwise first results of an international, registry-based, cohort study of Thoracic cancERs internAtional coVid 19 cOLlaboraTion (TERAVOLT) were reported by Garassino [8]. The study enrolled 200 patients with thoracic cancer and COVID-19 infection diagnosis since 26 March until 12 April 2020 in 21 countries around the world. To note, 147 (73.5%) patients were on treatment, 34 of which (23.1%) on ICIs and 20 (13.6%) on CHTs/ICIs combination. Preliminary analysis reported that no cancer treatment, including ICIs, was associated to an increased risk of death or hospitalization. Moreover, since it would seem that PD-1/PDL-1 pathway is an escape mechanism for some pathogens in preclinical models and the use of anti-PDL-1 could increase the clearance of some viruses, like influenza virus [9], it could be hypothesized that ICIs may play a protective role against SARS-CoV-2 infection.

In order to keep everybody safe, many cancer centers have reorganized the management of patients, avoiding gatherings and prioritizing treatments as well as according to scientific societies [1].

In our experience at Istituto Nazionale Tumori – IRCCS “Fondazione G. Pascale” in Naples, a tightened program of health surveillance for patients and healthcare providers has been planned and performed, in order to early detect and promptly quarantine subjects with SARS-CoV-2 infection. Reorganization of internal management has allowed to ensure an adequate protection for cancer patients afferent to our Institute. COVID-19 screening performed with rapid serological tests revealed that ICIs could protect cancer patients from SARS-CoV-2 infection.

## Materials and methods

From 30 March 2020 to 15 May 2020, 885 cancer patients admitted to Istituto Nazionale Tumori – IRCCS “Fondazione G. Pascale” in Naples (Italy) and 1050 healthcare providers working in the same hospital were tested for specific SARS-CoV-2 Immunoglobulins IgG and IgM, in accordance with a tightened program of internal health surveillance, addressed to ensure an adequate protection for frail cancer patients. In all cases entering the clinic during the period, the analyses were performed. Patients underwent a triage survey in which the proper questions to the symptoms were asked.

Patients’ cohorts included 287 subjects suffering from melanoma undergoing ICIs (anti-PD-1 or anti-CTLA-4) and 598 patients undergoing CHTs.

Patients with hematological malignancies were excluded, in order to avoid eventual false negative results, due to their state of immunodepression/immunosuppression. Their particular condition, in fact, associated with low titers of immunoglobulins, makes difficult for qualitative immunochromatographic tests to identify such low titers of specific immunoglobulins, albeit present.

Whole blood samples from patients and healthcare providers arrived to the Laboratory of Istituto Nazionale Tumori – IRCCS “Fondazione G. Pascale” Cancer Center and were centrifuged immediately. Plasma samples obtained after centrifugation were tested for specific SARS-CoV-2 Immunoglobulins IgG and IgM, using Leccurate – SARS-CoV-2 Antibody Test kit (Lepu Medical Technology – Beijing – Co., Ltd.), within 1 h, upon recommendations. The assay is a qualitative colloidal gold immunochromatography based on the principle of antigen–antibody reaction and immunoassay technique: it is a qualitative method for which data regarding cut-offs are not available to define positivity, nor related units of measurement, nor the detection limit. The producer declares that for the detection of sensitivity reference material, the positive detection rate should be no less than 90%; for the detection of negative reference material, the negative detection rate should be 100%; for the detection of positive reference material the positive detection rate should be 100%. The positivity to IgG and/or IgM is defined only visually, if the relative colored band appears near the areas where the relative anti-human mouse antibodies are pre-adhered, after binding any antibodies to a recombinant protein of the COVID-19 marked with colloidal gold particles. All tests were performed by the same laboratory technician and the results interpreted by the same operator, in order to avoid inter-operator and inter-observer variability. Both healthcare workers were blinded to group allocation of patients (ICIs and CHTs) and personnel. Patients and healthcare professionals were considered positive for SARS-CoV-2 infection if either IgG or IgM, or both, resulted positive at least at one determination. Data on age, cancer stage, Eastern Cooperative Oncology Group (ECOG) Performance score, line of treatment, symptoms, lymphopenia, leukopenia were collected for most of patients.

### Statistical analysis

Statistical analysis was performed by using the Statistical Package for Social Science (SPSS Inc., Chicago, IL, USA), version 26.0. Categorical variables were displayed as frequencies and the appropriate non-parametric tests (χ^2^ test) were used to assess significance of the differences between groups; p values < 0.05 were considered statistically significant. In order to assess association between cancer treatment (ICIs and CHTs), SARS-CoV-2 IgG and/or IgM seropositivity and clinical characteristics of patients’ sample—gender, dichotomized variables of age (less than 70 years versus equal or more than 70 years), cancer stage (IV versus I, II, III), ECOG performance status (0 versus 1, 2, 3, 4), lymphopenia and leukopenia conditions (yes or not), χ^2^ tests were performed and OR with 95% CI were computed.

A Propensity Score Matching was implemented in order to form two comparable subsets. Random choice of matched patients based on Nearest Neighbor Matching was implemented.

Multiple linear logistic regression was used to adjust treatment effect by other factors. A forward stepwise selection based on Wald statistics was adopted with 0.05 and 0.10 enter and remove significance level respectively.

## Results

885 cases with cancer diagnosis admitted to Istituto Nazionale Tumori – IRCCS “Fondazione G. Pascale” in Naples (Italy) from 30 March 2020 to 15 May 2020, undergoing different oncological treatments and 1050 healthcare providers were tested for SARS-CoV-2 serology (demographic data are reported in Table 1).

**Table 1:**
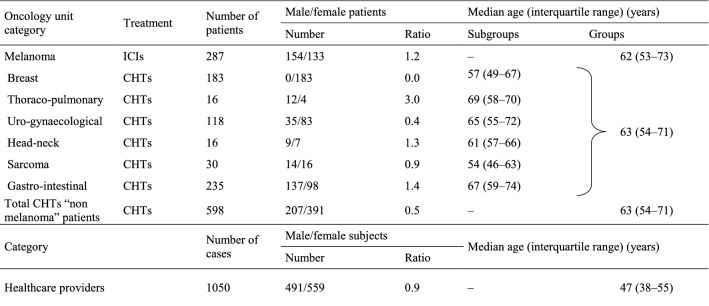
Patients’ and Healthcare providers’ demographic data

*ICIs* Immune Checkpoint Inhibitors Treatment, *CHTs* chemotherapy treatments

287 patients with a diagnosis of melanoma undergoing ICIs treatment (154 male/133 female) had a median age of 62 (IR—interquartile range: 53–73) years (Table 1). 598 CHTs patients came from breast (N = 183), thoraco-pulmonary (N = 16), uro-gynaecological (N = 118), head and neck (N = 16), sarcoma (N = 30), gastro-intestinal (N = 235) units, respectively, with an overall median age of 63 (IR: 54–71) years and an overall distribution of 207 male/391 female subjects (Table 1). 1050 healthcare providers (491 male/559 female) had a median age of 47 (IR: 38–55) years (Table 1).

Patients undergoing ICIs and CHTs carried out serological test for immunoglobulins. 9 (3.1%, 95% CI 1.1–5.1) of the patients treated with ICIs and 39 (6.5%, 95% CI 4.5–8.5) of the patients treated with CHTs had a positive serological test for immunoglobulins, p = 0.04 (Pearson’s χ^2^ test) (Table 2). At the same time, 1050 healthcare providers underwent serological test for immunoglobulins, 48 of which resulted positive (4.6%, 95% CI 3.3–5.9) (Table 2). The cohort of employees was compared with the cohort of patients undergoing ICIs and no significant difference was found (Pearson’s χ^2^ test) (Table 2). According to these data, we observed the higher incidence of immunoglobulins in patients treated with CHTs when compared to patients treated with ICIs or health care workers (albeit with some overlap in the confidence intervals).

**Table 2:** SARS-CoV-2 IgM and/or IgG positivity percentages of patients (divided into oncology units) and healthcare providers

Oncology unit category	Treatment	Number of patients	IgM and IgG negative		IgM and/or IgG positive		p-value
			Number of patients	Percentage of patients	Number of patients	Percentage of patients	
Melanoma	ICIs	287	278	96.9	9	3.1	–
Breast	CHTs	183	165	90.2	18	9.8	–
Thoraco-pulmonary	CHTs	16	15	93.8	1	6.3	
Uro-gynaecological	CHTs	118	114	96.6	4	3.4	
Head-neck	CHTs	16	16	100.0	0	0.0	
Sarcoma	CHTs	30	27	90.0	3	10.0	
Gastro-intestinal	CHTs	235	222	94.47	13	5.5	
Total CHTs “non melanoma” patients	CHTs	598	559	93.5	39	6.5	0.04*

Category	Number of cases	IgM and IgG negative		IgM and/or IgG positive		p-value
		Number of subjects	Percentage of subjects	Number of subjects	Percentage of subjects	
Healthcare providers	1050	1002	95.4	48	4.6	0.29**

*ICIs* Immune Checkpoint Inhibitors Treatment, *CHTs* chemotherapy treatments

^*^Comparison ICIs vs. CHTs patients’ SARS-CoV-2 IgM and/or IgG positivity percentages (statistically significant) (Pearson’s χ^2^ test was used to identify differences in the proportions of individuals between two categories)

^**^Comparison ICIs patients’ vs. Healthcare Providers’ SARS-CoV-2 IgM and/or IgG positivity percentages (not statistically significant) (Pearson’s χ^2^ test was used to identify differences in the proportions of individuals between two categories)

In the analysis of association all the other clinical variables were significantly associated to cancer treatment except age (Table 3), while SARS-CoV-2 IgG and/or IgM positivity was associated with gender (p < 0.001) and cancer stage (p = 0.03) (Table 4). Data about symptoms recorded in the triage survey did not present relevant statistical differences between the cohort of patients.

**Table 3:** Association between type of treatment and clinical variables

Clinical variables	ICIs	CHTs	n	p-value^*^
SARS-CoV2 IgG/IgM				
IgG and/or IgM+	9	39	885	0.04
IgG and IgM−	278	559		
Gender				
Male	154	207	885	< 0.001
Female	133	391		
Age (years)				
< 70	194	416	885	n.s
≥ 70	93	182		
Cancer stage				
I–III	38	97	804	0.05
IV	249	420		
ECOG performance status				
0	244	265	793	< 0.001
1–4	41	243		
Lymphopenia				
Yes	2	90	755	< 0.001
No	256	407		
Leukopenia				
Yes	0	91	764	< 0.001
No	258	415		

*ICIs* Immune Checkpoint Inhibitors Treatment, *CHTs* chemotherapy treatments, *n* number of patients, *n.s* not statistically significant

^*^The p-values represent χ^2^ tests of independence indicating associations between type of treatment and categorical clinical variables (statistically significant p < 0.05)

**Table 4:** Association between IgG and/or IgM seropositivity and clinical variables

Main features	IgG and/or IgM+	IgG and IgM−	n	p-value*	OR	95% CI
Gender						
Male	11	350	885	0.01	0.41	0.21–0.82
Female	37	487				
Age (years)						
< 70	31	579	885	n.s	1.23	0.67–2.26
≥ 70	17	258				
Cancer stage						
0–III	12	123	804	0.04	0.48	0.24–0.97
IV	30	639				
ECOG performance status						
0	25	484	793	n.s	1.16	0.61–2.20
1–4	16	268				
Lymphopenia						
Yes	5	87	755	n.s	1.06	0.41–2.79
No	34	629				
Leukopenia						
Yes	6	85	764	n.s	1.38	0.56–3.36
No	33	640				

*n* number of patients, *OR* odds ratio, *n.s* not statistically significant

*The p-values represent χ^2^ tests of independence indicating associations between SARS-CoV-2 IgG and/or IgM seropositivity and categorical clinical variables (statistically significant p < 0.05)

To better investigate the association between cancer treatment and seropositivity, we performed a Propensity Score Matching based on gender and tumor stage. We obtained two groups of equal size (n = 287, each) in which sex and tumor stage were perfectly balanced. The effect of treatment on seropositivity in this matched subset was analyzed through a regression logistic model and the ICIs treatment resulted to be the only protective factor significantly (p = 0.03) associated with positivity (OR: 0.41; 95% CI 0.18–0.91) together with female gender being a significant (p = 0.01) unfavorable item (OR: 2.87; 95% CI 1.29–6.41).

## Discussion

The concept of frailty has become increasingly recognized as one of the most important issues in health care and health outcomes and is of particular importance in patients with cancer. Frailty is a complex state of diminished physiologic reserve that results in increased vulnerability to stressors, leading to adverse health outcomes [10]. One important clinical question is how to manage patients who need anticancer therapy, including ICIs during these conditions.

As reported by Poortmans, the more recent larger studies have evidenced that none of the anti-cancer therapeutic regimens may affect neither the rate of severe COVID-19 nor the mortality rate in cancer patients [11]. On the contrary, the study conducted by Robilotti at the Memorial Sloan Kettering Cancer Center, has highlighted, specifically for ICIs, an association with increased intensive care unit admission rate, but not death rate [7]. So, Vivarelli et al. conclude that the question remains still debated: is ICIs administration harmful or beneficial for cancer patients during the COVID-19 pandemic [12]? Their hypothesis is that using ICIs in cancer patients during the pandemic does not harm and might be a game-changer. Based on the positive effect that ICIs have towards T-cell reactivation against cancer cells, as well as virus-infected cells, they conclude that ICIs administration may not represent a risk for cancer patients during this pandemic and can be suggested as protective for cancer patients who are infected by the SARS-CoV-2 [12]. According to this hypothesis, our preliminary data suggest a possible protective effect of ICIs against SARS-CoV-2 infection onset. In our patients’ cohorts, individuals undergoing treatment with ICIs presented significant lower proportion of IgG and/or IgM positivity in respect to patients treated with CHTs, ICIs seeming to be a protective factor against COVID-19 infection. At the same time, ICIs cohort presented a risk of SARS-CoV-2 infection similar to that of healthcare providers, showing that ICIs treatment is able to restore an adequate immunocompetent status in cancer patients. When a Propensity Score Matching based on gender and tumor stage was performed and effect of treatment on seropositivity analyzed, the ICIs treatment was demonstrated to be a significantly protective factor.

Overall, according to our findings, we may carry forward our hypothesis according which Innovative Immunotherapy based on ICIs treatment could protect cancer patients from COVID-19 infection. Nevertheless, further studies and epidemiologic data are needed to better define risk population among cancer patients and fill clinical and preclinical gaps to provide strong evidence-based therapeutic guidance and physiopathological insights on the possible immune intersection between COVID-19 disease and cancer therapy.

A registered clinical study currently ongoing (NCT04343144) conducted on severe patients affected by COVID-19 requiring hospitalization in conventional unit or in intensive care unit will give us additional insights about the possible role of ICIs in protection from SARS-CoV-2 infection, uncovering the differential efficacy to eradicate infection in COVID-19 patients treated either with anti-PD-1 antibody nivolumab in association with standard care protocol, or with standard care alone.

## Data Availability

The datasets generated and analysed during the current study are available in the Vivli repository, 10.25934/00006101.

## Abbreviations

COVID-19: Coronavirus disease 2019
SARS-CoV-2: Severe acute respiratory syndrome coronavirus 2
ICIs: Immune Checkpoint Inhibitors
CHTs: Standard Chemotherapies
OR: Odds ratio
95% CI: 95% Confidence intervals

## References

[1] van de Haar J, Hoes LR, Coles CE (2020). Caring for patients with cancer in the COVID-19 era. Nat Med.

[2] Desai A, Sachdeva S, Parekh T (2020). COVID-19 and cancer: lessons from a pooled meta-analysis. JCO Glob Oncol.

[3] Barlesi F, Foulon S, Bayle A, et al. Outcome of cancer patients infected with COVID-19, including toxicity of cancer treatments. In: AACR annual meeting I, April 27–28; 2020.

[4] Rugge M, Zorzi M, Guzzinati S (2020). SARS-CoV-2 infection in the Italian Veneto region: adverse outcomes in patients with cancer. Nat Cancer.

[5] Liang W, Guan W, Chen R (2020). Cancer patients in SARS-CoV-2 infection: a nationwide analysis in China. Lancet Oncol.

[6] Zhang L, Zhu F, Xie L (2020). Clinical characteristics of COVID-19 infected cancer patients: a retrospective case study in three hospitals within Wuhan, China. Ann Oncol.

[7] Robilotti EV, Babady NE, Mead PA (2020). Determinants of COVID-19 disease severity in patients with cancer. Nat Med.

[8] Garassino MC, Whisenant JG, Huang LC (2020). COVID-19 in patients with thoracic malignancies (TERAVOLT): first results of an international, registry-based, cohort study. Lancet Oncol.

[9] Rutigliano JA, Sharma S, Morris MY (2020). Highly pathological influenza A virus infection is associated with augmented expression of PD-1 by functionally compromised virus-specific CD8+ T cells. J Virol.

[10] Ethun CG, Bilen MA, Jani AB (2017). Frailty and cancer: implications for oncology surgery, medical oncology, and radiation oncology. CA Cancer J Clin.

[11] Poortmans PM, Guarneri V, Cardoso M-J (2020). Cancer and COVID-19: what do we really know?. Lancet.

[12] Vivarelli S, Falzone L, Grillo CM (2020). Cancer management during COVID-19 pandemic: is immune checkpoint inhibitors-based immunotherapy harmful or beneficial?. Cancers.

